# Supplementary figures and images for: Diagnostically relevant facial gestalt information from ordinary photos
Source: eLife. 2014 Jun 24;3:e02020. doi: 10.7554/eLife.02020 (PMC4067075; doi:10.7554/eLife.02020)

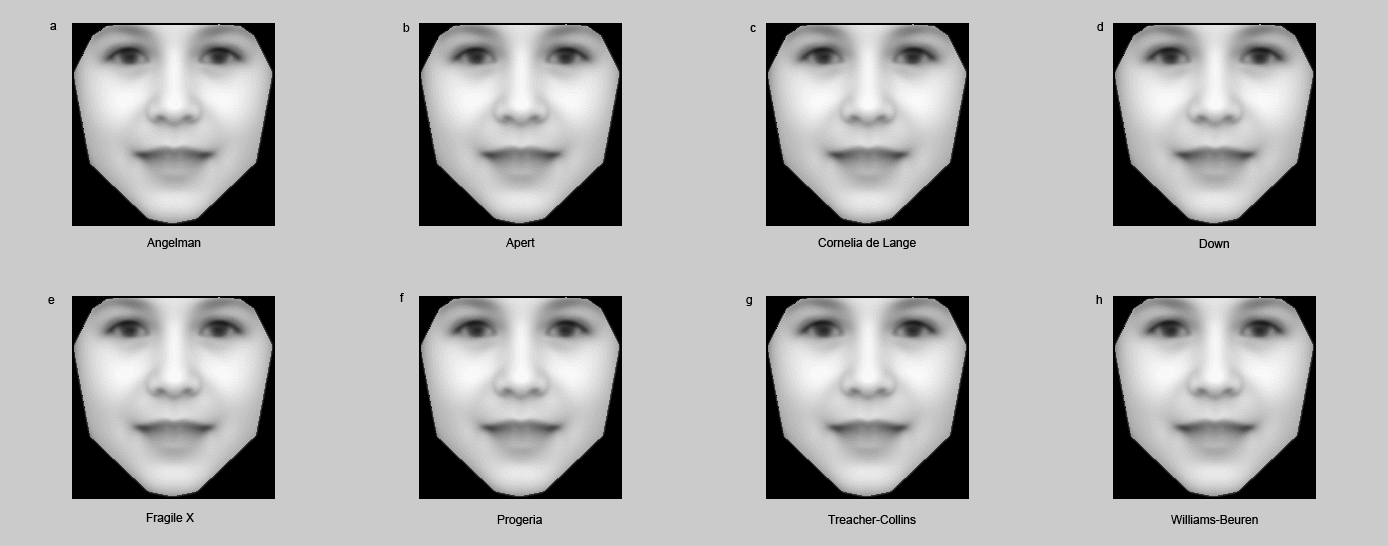

Supplement: Supplementary file 1 [file elife-02020-media1.gif]
