# Supplementary material for: Diagnostically relevant facial gestalt information from ordinary photos
Source: eLife. 2014 Jun 24;3:e02020. doi: 10.7554/eLife.02020 (PMC4067075; doi:10.7554/eLife.02020)
Supplement: Supplementary file 1. — Tinyurl links to sources for the database. Prefix the 7 characters with http://tinyurl.com/. Links are expected to decay with time; the full dataset will be released to researchers at the discretion of a Data Access Committee. DOI:http://dx.doi.org/10.7554/eLife.02020.007 [file elife-02020-supp1.doc]

**Table 2.** Tinyurl links to sources for the database. Prefix the 7 characters with <http://tinyurl.com/> . Links are expected to decay with time; the full dataset will be released to researchers at the discretion of a Data Access Committee.

| **Angelman** | |  |  |  |  |  |  |
| --- | --- | --- | --- | --- | --- | --- | --- |
| qy7rs5q | ozc2sjh | peh2nng | olc3r3u | og7bk73 | nv75tyo | nnumdpg | phh3m7t |
| pkh4kp9 | q77tp7j | nzpu6sm | pmlst4n | q535gs5 | qehd7gr | pcqgadz | nlpv5d2 |
| oq2o5v8 | nh2aqzo | p7lo3go | qh39avo | pocf9el | o8gn9sc | oeuuywl | qzcnt66 |
| o8us4us | qxcz5xr | q234zap | pxc7l7g | otvyrkk | oslk9d5 | q3qnvkn | p9cagqm |
| ns74uhb | pjlruka | phv8hac | o5ur6df | pqvbctz | nbuzjlu | pj7n2hr | ob2jda9 |
| nlbqaaj | ndl4wvs | o2uq7t2 | pe2uslx | nobr8rw | ps7pv4x | p22ryhe | nw2fj6n |
| pa7k6vl | p2gvtju | o477q3h | q8hbafy | pd7l5gy | o7ldjnd | qfcmulq | p6c9z99 |
| q4mxlpm | pg7m4xe | ohuvxhy | nzbpbq6 | ozq7nmx | pth7gbf | p3qderc | pv7qtld |
| o22gho3 | klkvbyo | ngl5vg8 | ocq5qk7 | q7myka2 | nqunc9t | obut3f8 | qxqnx3a |
| **Apert** |  |  |  |  |  |  |  |
| oosgzuc | nqprtf7 | pawc6jg | perms8s | pzyq7rg | p4nu35u | nvhqxk3 | q7zgtpv |
| 2v6zpp9 | nqprtf7 | oghvopf | nj3hbey | onkcqxf | oa9glbt | qesxc26 | qxn59j6 |
| p25xwox | njug98f | o2r8hbv | p7wb823 | nmaknwm | pzekaje | nectwjn | qxn59j6 |
| o7nkhtw | oebto2g | o2r8hbv | nwr7zrz | ogpy5qn | oox6spd | o8raedo | qedsgwn |
| pfso74h | q37nknz | n9m9kpc | plnyffk | pou4sy8 | nmneqok | ns3k7x7 | ndvp4h7 |
| p9xcjup | q7h64fd | ovz3hde | qg4eu9l | n9tcyqk | oh4s8zl | omzxlr8 | p2rjy59 |
| oezw53o | ndg9h46 | o43n2jd | p4z5ef7 | pq6jd8n | q6xm56n | nl88jqf | o8d5jb8 |
| q3eqyoq | npgdb7p | nncws5t | oa3owl6 | nnk2974 | qjocck7 | ofmmykg | pu8wfwa |
| oxs8un7 | pec77sr | ohrd9wu | q3yvvws | pcft5s6 | ovgpp2t | q3n683j | nrmfbuo |
| os9mch8 | q8qkmms | q6n76kw | nb5qky2 | ozyfoy5 | ppv66tu | o68eavr | pgz98zq |
| qh5g95a | ns29emm | nghl7w4 | prgxvg5 | p5khzle | nnbm2sb | p7z6cwk | nkr3onw |
| nbzkmqx | pbqb4du | pwrsje7 | nzy577r | nufdtdt | q6lteae | pl8tkd4 | p9mulcy |
| poem7l7 | ohbumjt | qdzzqro | ocfzmys | qdpl7sw | p5c4c9l | qfna275 | pndkspz |
| noey7yz | o3l7f6c | nd3fec8 | qybczmu | p26bpyz | pwqhr3m | nf86mom | osw7dvh |
| q7o8zgl | ov2kuxd | ol8j3jq | pkkmb9d | ptkp6sj | oole4t4 | o9mk4zn | qhsyajj |
| ocx3ylr | qjveqlf | np3j8fr | qf22q3e | p9ync8a | nd24myn | nsvtu56 | py3wksb |
| qxstv9t | ov3vmav | nz87l92 | o9ycsew | oek9ue9 | o7gz3so | ondabw7 | npvsvkq |
| pk56ntc | ppzc44w | ocml333 | ph6ghmh | oek9ue9 | p9xcjup | ooyhl2v | odnmevp |
| opnq92b | nmvsx3d | nvvusmj | ph6ghmh | osbx4rq | oxs8un7 | q6ywuh8 | plsq46a |
| ne4gq7u | q692bzg | pkropal | pab4ufp | qh6ryfs | qapk9bj | qjpn4up | oc8g8xk |
| o8zu8yv | oahtrnm | qf9574m | q4pzc9q | pru5qzl | nkkyamo | ng7y3dp | oj3rs7c |
| pueo4nx | qd4dvp8 | neryrm6 | poys4t9 | ojp24b3 | p9fs7aq | pwh8ess | no89zas |
| pgoxu9g | o73oy3q | pv49649 | ow6aqh5 | p7b3vvc | q3u8m4r | ooc4pla | ozmnx4t |
| qb5ec3h | pf8rnbb | phdzvnp | ozfkj3l | qb6p4e2 | oyaoz38 | ncps9qt | oltqcnh |
| p9sma2o | q9n855c | qbrwdhq | q7befon | oyotd5o | pn6zeoa | o778oku | o7nkhtw |
| ofe8s8n | njhm6gh | nlmdesv | obk8vtt | ot69rwp | ncy3a6y | nqgzh7d | oaardme |
| pnjchg8 | odw2laz | paz7bhx | qxg2shv | qz23okr | pxtu6r3 | p8hxqln | pw6laag |
| ncducuw | ox8cduy | og3qtmw | pjpcl4e | nq5vcmy | pekke7k | pm7oy27 | o9tmzjv |
| py9o9jy | qgzjpc4 | q4wkqby | p2kgk4y | q9g5o44 | q92xtyl | orqaj86 | pvpgf8x |
| pfxegwz | navo5wr | po8uzuh | o8635ax | njajpf9 | nwk54qa | nb28tgv | p6qedbp |
| nj99x4o | owrz2kt | owdd7zd | puyuyv2 | q2kr3uc | oryzjkb | nopw3uf |  |
| odnmevp | pr8vheu | o4vwpoc | pgb6rhz | p4p7sgf | oryzjkb | oj7czog |  |
| **Cornelia de Lange** | |  |  |  |  |  |  |
| qcr8dld | ogtxfpb | oha5arr | qj6mrxk | q2fvl2n | ndsmxek | pyfybs7 | okybgzu |
| nmlmewz | ppy44wt | o3jf3ea | o45uxpw | nfozgxo | omxpmm5 | okob4en | nzojez4 |
| oh32tqj | naxbbp8 | n9abntf | ohtk86g | p4kcclq | qatjja7 | njej2du | od5wtc5 |
| q5vtqel | o4eoqj9 | o3op5ng | ngjs65n | pwyprfr | p9jrh9e | odswh7v | plb3zjn |
| pp86jy4 | nnoyj5p | pbtsrvy | oby8kwo | ntf8ne8 | ost49su | qgfgl9h | p467hja |
| n9j6gmp | oz5fxwq | ng5nb37 | pj6ca79 | ontm589 | nfj8doz | nto3g7z | qcbb6qs |
| o4xjvgq | p5ogsk2 | oak3pu8 | ooovsd8 | qcpgytb | pz5phq4 | oo5gvyj | ogk5mwy |
| pc5nkob | pjf73xj | pzp7f4q | o7k2rcs | pbfnwsz | qck5wk5 | nmsotxq | pop9c6j |
| ohoa5ua | ndeh5c4 | ncohhgb | pfp6gkd | q5u4emj | pd6ad5g | ochpk8e | pnymvtl |
| p5acxhj | o7suk65 | ptyosve | p8trtdl | o3akalx | q7knrwh | qydjgtg | nbt7rbc |
| pwopfbk | pgyy9dn | nqf7ots | oxoo763 | qjkrm33 | p6avpvo | oq8frlv | o6al95d |
| qdffmp4 | oljkrjm | q9pf3bv | nbf2w8t | o3vrjoo | qxpc7pp | p3zx8fj | npjvyot |
| ptajloo | oeo97cu | njjt4n3 | prpaanw | pc3v8w9 | qeg2f69 | puwdn9k | pob4h43 |
| ol5fwh6 | peaet3p | p6p3ly7 | np5q6md | ohmhr58 | o8fchhr | nsctj8y | qzphvv4 |
| qay9t58 | odxmr2w | oqyddkn | nzaejfl | p58jlph | pgkg6pc | omray22 | najq93u |
| q35v8vg | nw9wjmf | nef3uq9 | p768g3n | oar66vg | q9ba89f | qbn3un4 | owfk5ow |
| pmf8yhw | p4xufa4 | p5tqut8 | qykluuo | pwnx3jz | nenvnjj | puky6w6 | orw27vt |
| osxrjnv | pvfau45 | pjkh59p | pktvnh7 | pzw9t6y | phafrl5 | qbaoecn |  |
| nb9ptwn | phokmnl | p2fl49c | oqfz8n6 | qddnbx2 | nljaaqb | ofjzuht |  |
| npxg4d7 | nmxf5sr | ptuyj3d | qfbc5b8 | pyrds5l | opevg9y | ota94vd |  |
| q8anfsa | pat92gt | pg6bbmt | o9am7mq | nqma5v3 | pkoll8y | opt3bbh |  |
| p7xvdrh | ojxoo4p | oloutss | phfptub | pfz42j5 | qf62322 | o8o7ab4 |  |
| qyfbslz | nvemthg | nkf5rs2 | qbu6boc | njqvzob | nlokd2h | o2fakgy |  |
| oe8cxzc | q3pd6a5 | oxhlq4s | oetj9m3 | nz3c5ed | pdrhm97 | nb8xz7l |  |
| **Down** |  |  |  |  |  |  |  |
| 676mj7c | ct2c64c | kflerk3 | c5zyoop | q7jrm3g | cwumrqo | crtfbey | c3682b3 |
| mra8pee | bl7slf6 | c8js2y7 | lmo6cle | p6a2k2n | calyjth | cgol7fe | mvhhawn |
| m2eoe2a | bvhd28r | l8c8t46 | mutaysw | nv4vus5 | dyl983m | kuk8ruh | cwb7uf2 |
| oeyb9w5 | ckrya6y | lf7kgav | lfsrpem | pkxprg7 | c9potoz | dxth8fr | c8bx97u |
| lwxzkfe | d8zd72g | bsyettt | nyafsfs | opjccm6 | ck29az6 | kyf8wqz | csu23s6 |
| mhtyypp | l78zwke | kqq8vzn | llstmgf | qef69a8 | ck29az6 | l2pyyrb | kxe7kut |
| kznmob2 | ktwyzqq | cdtub77 | k6sed25 | o8egblq | d9buzfd | cltdec8 | mnjqjjo |
| mf32h9k | mbwgs83 | m5czed4 | lpa2g3b | om56j2a | cqgg2mf | d2k3lpp | d9kpb8n |
| 3q7tl2m | l23qpnk | knc34vr | kfecdzs | p9o8dmj | d776a2w | lmtvyc4 | kanpyoh |
| 6cfgpq3 | lfrhx44 | m3lwy36 | mot84r6 | of9rvsz | bquadvk | nyg7h6z | ctz9cvu |
| ke74qhw | lcdb6h8 | m3lwy36 | 67a9oj7 | ocvy9et | bmkru6p | l8av3ql | m5jksec |
| c2nasgz | n84gnc2 | m3lwy36 | ca8m3j3 | qfhzdgf | ca6tory | bpa8o5q | d3dlx5m |
| c9zmfna | kdmf7vc | m3lwy36 | kj65bo3 | nsq58eg | n5wpewl | czy2pmt | n55qh57 |
| lxutvx9 | kh3lyz8 | m3lwy36 | luperzh | nluql7o | cbug53w | ch5q2hu | cj8ow59 |
| cqnzgnn | mcrqzd2 | m3lwy36 | l8fqjla | pohap9a | nxsx82r | d86xnp2 | myoa6px |
| d5nmar9 | cm8pumm | kg2jmmj | caygu8m | pr36so7 | c8gabh3 | mshgcfa | m55gxbt |
| cxwof6k | mb47534 | c9kerfc | o7j6lgr | qxvzfuw | o5sk86t | qfafwf7 | q2m4t7u |
| c296xe2 | ld8ktm7 | c669xsg | 4yjt5s9 | bcqqooc | proe4rv | qbta5sb | plvelrd |
| bsdrd7a | cghzqe6 | k7glm5t | nqsge3a | p32w7h2 | pexnuee | og54mxh | nfuoo5v |
| cje2yef | c2qsjxm | cdykl28 | pfoaaoc | o8lzpny | 7ef3uc7 | o2eeekx |  |
| chnfl4h | cncudkv | m6lwwkj | p8svnhk | pgqmeuj | p75ca7m | nazu37t |  |
| cl4leod | cz856o4 | d7gy2t9 | crntcnh | omc9x3z | ocnvsdl | owenwsv |  |
| cpe3vg8 | d73ljzs | d2b8swf | os58g43 | nl2gv2p | qyjppyn | pqt4emw |  |
| clwv4tc | buxt3x4 | ctpbrx5 | qhy2cp5 | ofguctq | pkt2hl6 | no9kald |  |
| d3bsldk | d55wp7m | cjfrd6h | crzrdzs | pnmy24b | ncgzuj2 | 25xauvq |  |
| pyccr74 | o6uwcd3 | pf33ykl | yhjq6ps | oklmjqk | 25xauvq | p87oedt |  |
| **Fragile X** |  |  |  |  |  |  |  |
| d2ecx22 | brufjv8 | d7lpojw | br6jq6e | bpn2quo | ccgggy4 | d9jezv5 | boknmoh |
| d99nk8r | cypr75x | bp75k36 | d2bnfdw | c6dqyb8 | bu26sam | c22z5rb | cpufopa |
| ctsev2p | bozvawf | ccq7enf | csgbs77 | bwzee4g | d8lgb53 | cmlkxek | c3krgs3 |
| cjzuh94 | cwo2x7x | cvlmtxq | cmueq7u | cd94lzx | c328pcf | cj7e6ro | dygdp9x |
| cqe94ft | cc5x5jo | bqupxd4 | bnb94h2 | d7nz3cy | cluo6mq | bvlcc7f | bmyrj9k |
| chsb3w6 | dyeldgv | cs7h2du | bqpev3v | bnwgdkq | c9gfhgo | d6qgyfx | c2phqn4 |
| cvjybcn | cu55tp3 | ch2mvfa | dyuzkbg | czdtser | cculb3k | coc6doz | cnbjlad |
| bu999bu | bpe8y4e | cuqd5sr | cs27x4o | bursxrz | d5lfckn | c77aco5 | c9ktdtt |
| cawwgpe | d7enazo | d4loq2j | cnpogct | d5wvn23 | d92l67j | cngtnjj | cqppeu9 |
| chxydr7 | cz522lh | crc7c8v | ckbzmqx | brdm77m | c6ujeyr | bplaf5m | bnpdwjz |
| ca4mqkf | br4reec | bobstv7 | cr6u9wp | d2zptf7 | d87bg2k | ck2ntwn | cbbfr6r |
| cke77e3 | d29v3lu | bv77h4w | d7ukhd9 | cy9xree | cwwtqxa | codvpgk | bouelds |
| bws5cqr | blzucf2 | cplkvwx | cpgatmr | cewoyrv | bvn4nwh | bqzch2n | c89mguc |
| d8x92yc | d3jcltc | d2v5cpl | brkol8u | blrp58c | ccdrvdy | cxdo49f | c8pjnou |
| cwebyhm | d8o4sq6 | cfc3z5c | csugn9n | bsn3pf4 | d6s8d72 | cuexmzd | cj96zjq |
| c8e3dah | cax8u7a | c8rc2gw | bldka5t | c5wl68p | bn46nfr | bt48khk | c7n7jzn |
| cyzoq3p | cdeenr6 | cj3tgak | cfsxowu | ccrwqfh | bmnysdb | c9rvrv4 | ckzl3r8 |
| cfum9lx | czj6uox | cpp5mg4 | c24ahjd | cos3kj3 | cnwque4 | dy9bb7p | d84mveh |
| bsbkfxo | bvx533o | cyu83z5 | d3e2jk6 | clduqw7 | d4nh4rl | cdn9gkg |  |
| **Marfan** |  |  |  |  |  |  |  |
| clad3f3 | bu33y6n | cr3cjfl | cyczhvz | bpcfmcc | bscgktp | d3vp56q |  |
| bqxmvg3 | c7ckfxb | cw84qcf | blv9spg | cjqt46d | bpqkhes | d7cuvqm |  |
| c2mx36x | dxhjbju | c87u55a | ce7w273 | crafxgt | cbm2vp6 |  |  |
| **Progeria** |  |  |  |  |  |  |  |
| orcjcms | nvyp5y | ofrhhf5 | ptwn3og | o48zze2 | pzrswpt | qy95kgb | nh3lzt9 |
| 8xn2v28 | pfdn66x | q4nbe37 | olrjegv | nnhsbxz | pzrswpt | o8w5u8d | qxdsubc |
| o3rdnbj | cfep2qf | pnwl6mn | olrjegv | cnvuoz3 | qd99eju | ns8fmrt | pjn5mus |
| pbwhcj4 | q9s3lxy | qzeylgo | oy8hjtm | pwwo28t | qd99eju | ph49jfv | qe4j4ot |
| bluusod | p8zbzw8 | ow43saj | bpf8vsr | nud6v7a | o78jgvf | ommt4lu |  |
| q6durd5 | oxd7ton | pqzha4k | nbvtbwf | qdne9md | o9rfkdc | n9r53ky |  |
| q6durd5 | 394ezx | nnwx63y | a6cnnlc | qdne9md | nt3pcws | q3dtssp |  |
| oampazb | nor9t8x | ozddku2 | npmjjcw | o7mocxv | ogmr7k4 | os8q6l7 |  |
| ntw234r | nhvv9y8 | q797hz4 | od8lex8 | nk3mhdm | q5zl486 | ptwn3og |  |
| **Sotos** |  |  |  |  |  |  |  |
| 9ab2xv6 | cnwuzqx | cfdwz9a | cbf462r | cntcs8t | cxrwknr | bv93h8u | cqy43la |
| bw4d7dt | c6syhpk | c2xtfe5 | cskx737 | boa76zy | btzh3vw | brrunmy | d8fdobp |
| cvnzt4o | d84qjqd | d3alhev | cqf8vnq | cgqvrxt | d8tzje8 | chtav73 | czhz7ar |
| **Turner** |  |  |  |  |  |  |  |
| d6e3z4j | bla2jlp | bqerqzj | cd5jvxt | blo7eo8 | ccof2vd | d2f85v3 | d45rj8y |
| cm97h46 | cqyxf9e | d8fa2wt | d5tdwhw | cnxwgpy | bua5f7v | cjjqn55 | coaeywg |
| d9s9bof |  |  |  |  |  |  |  |
| **Williams-Beuren** | |  |  |  |  |  |  |
| ptbfrkp | o5aumk9 | o5p2hmp | pppt4ko | nnv4x7x | nhanwuh | nwdha6j | pes4enc |
| nqovnj6 | odwh7p2 | qf9jqj6 | pppt4ko | pcquts2 | p66htzj | nf8l966 | q99hthd |
| ol6c5d7 | qgjybql | nerge3n | nn6cxj5 | ozchfyy | qxkvbck | p44f5r8 | q99hthd |
| qa262y9 | naw5peb | o98tsuo | nn6cxj5 | nh2pdx8 | pju83w3 | p44f5r8 | p7wprgk |
| p8pexxg | o5dj78c | nsz6lh7 | nn6cxj5 | qxcwofb | ndtz6al | qc9zsyq | p7wprgk |
| q36rerh | oj49elt | omwk3b8 | q6gjcy8 | pjm9hyr | o8avl4m | nm3wtcv | nwrm592 |
| pmg47ex | nhrhcl3 | nlmsyaf | p56raxf | qe3mvss | pgg2ac7 | ogzdb6w | plngyu4 |
| qguhm8y | oc8urf4 | pazluvh | pwl7org | o83xy7d | qaufp68 | p5dtoyn | plngyu4 |
| nbam2so | qz9kp3j | po9c5cy | oog42jv | olqn8ku | nkotqhd | pj4jvf7 | nfmq48m |
| o5p2hmp | ovwmvue | nazxubs | nctrjph | nkgv6k4 | of6a8be | oon6gl6 | oa46j2n |
| npyca98 | pcnd6av | owdrqwu | pyplgdj | oy7lexl | oyfkxuu | nd3uxqp | ntdgbm6 |
| ojfpp39 | obdl4a5 | pqs88qv | qabuxnk | qhhspfo | osuxfov | o7zafkq | qcnnn49 |
| q8bjlob | op4bbnm | pd4zyde | ogbau5o | nplndgv | pyypmlp | pte4c8s | qcnnn49 |
| pkphr42 | qex58bo | q7zvg7f | pogejd9 | ok33saw | nptmxe7 | np4xrub | q5sc2uh |
| pv227un | ndzyst8 | qxnjtxn | o56gq74 | nzqbqa6 | q96tbta | q8e86ce | q5sc2uh |
| pv227un | p2dspha | pjwtlk6 | pk6w2le | od7p847 | odfnsyg | nz8m7nj | oun8dmw |
| pv227un | o7wfan9 | p2sxkjq | ppmbe3k | nwh2xnn | nhv2357 | pe3cf2h | nlqcorj |
| nlk6gmc | pts97bb | nrmtvc8 | ncq9t8d | plcsubp | occfhw8 | ojlwx8g | og7q6lk |
| nlk6gmc | nr8p39p | pgznsxa | nqhx3lu | nqv4vod | pkhj76q | q8hpttz | qzraan6 |
| q4uds4f | pg4zwur | qbx49rb | q2vsru8 | qa8ca6g | oq35pcp | p78xrsp | nhgu82o |
| otpa5tu | qazweos | p9nc7qz | orqp5mn | oeh6qbm | negt9zb | qyme9mq | qxr4jhr |
| nafccnp | p997co2 | oyz8zjx | nb2nfvf | q3dxmwo | p3cn66d | nw2u5l7 |  |
| o4tprhq | q3nkrz3 | pne2f72 | prcurdz | pmmafj7 | pur3lxe | oqhakf8 |  |
| pqpjo5s | oy43ngh | qhsfuy3 | noqdnbw | nbgsbxv | ph3ddju | p3qsy8t |  |
| no62k49 | pxn9c7c | o6nxpfr | q83lyr2 | o5v8prw | qbhqsdv | o9cejes |  |
| ozkgyva | ojwj4qs | npwaz2a | noqdnbw | prr2mgy | nlc7tp3 | nslocyb |  |
| pa8yqd5 | q4mf876 | othbzwl | qzd5fkn | na7dpqg | o4lr7kh | on34qsc |  |
| **22q11** |  |  |  |  |  |  |  |
| njentvd | orjrj5v | p4tbwwj | nto8apy | oo5lpj2 | pbonk6b | o3jjuvq | o3oux7w |
| ppkntty |  |  |  |  |  |  |  |
